# Building a comprehensive cardiovascular magnetic resonance exam on a commercial 0.55 T system: A pictorial essay on potential applications

**DOI:** 10.3389/fcvm.2023.1120982

**Published:** 2023-03-01

**Authors:** Juliet Varghese, Ning Jin, Daniel Giese, Chong Chen, Yingmin Liu, Yue Pan, Nikita Nair, Mahmoud T. Shalaan, Mahmood Khan, Matthew S. Tong, Rizwan Ahmad, Yuchi Han, Orlando P. Simonetti

**Affiliations:** ^1^Department of Biomedical Engineering, The Ohio State University, Columbus, OH, United States; ^2^Cardiovascular MR R&D, Siemens Medical Solutions USA, Malvern, PA, United States; ^3^Magnetic Resonance, Siemens Healthcare, Erlangen, Germany; ^4^Institute of Radiology, Friedrich-Alexander-Universität Erlangen-Nürnberg (FAU), University Hospital Erlangen, Erlangen, Germany; ^5^Dorothy M. Davis Heart and Lung Research Institute, The Ohio State University, Columbus, OH, United States; ^6^Department of Emergency Medicine, The Ohio State University, Columbus, OH, United States; ^7^Division of Cardiovascular Medicine, Department of Internal Medicine, The Ohio State University, Columbus, OH, United States; ^8^Department of Radiology, The Ohio State University, Columbus, OH, United States

**Keywords:** CMR, low-field, 0.55 T, cine, flow, LGE, MRA

## Abstract

**Background:**

Contemporary advances in low-field magnetic resonance imaging systems can potentially widen access to cardiovascular magnetic resonance (CMR) imaging. We present our initial experience in building a comprehensive CMR protocol on a commercial 0.55 T system with a gradient performance of 26 mT/m amplitude and 45 T/m/s slew rate. To achieve sufficient image quality, we adapted standard imaging techniques when possible, and implemented compressed-sensing (CS) based techniques when needed in an effort to compensate for the inherently low signal-to-noise ratio at lower field strength.

**Methods:**

A prototype CMR exam was built on an 80 cm, ultra-wide bore commercial 0.55 T MR system. Implementation of all components aimed to overcome the inherently lower signal of low-field and the relatively longer echo and repetition times owing to the slower gradients. CS-based breath-held and real-time cine imaging was built utilizing high acceleration rates to meet nominal spatial and temporal resolution recommendations. Similarly, CS 2D phase-contrast cine was implemented for flow. Dark-blood turbo spin echo sequences with deep learning based denoising were implemented for morphology assessment. Magnetization-prepared single-shot myocardial mapping techniques incorporated additional source images. CS-based dynamic contrast-enhanced imaging was implemented for myocardial perfusion and 3D MR angiography. Non-contrast 3D MR angiography was built with electrocardiogram-triggered, navigator-gated magnetization-prepared methods. Late gadolinium enhanced (LGE) tissue characterization methods included breath-held segmented and free-breathing single-shot imaging with motion correction and averaging using an increased number of source images. Proof-of-concept was demonstrated through porcine infarct model, healthy volunteer, and patient scans.

**Results:**

Reasonable image quality was demonstrated for cardiovascular structure, function, flow, and LGE assessment. Low-field afforded utilization of higher flip angles for cine and MR angiography. CS-based techniques were able to overcome gradient speed limitations and meet spatial and temporal resolution recommendations with imaging times comparable to higher performance scanners. Tissue mapping and perfusion imaging require further development.

**Conclusion:**

We implemented cardiac applications demonstrating the potential for comprehensive CMR on a novel commercial 0.55 T system. Further development and validation studies are needed before this technology can be applied clinically.

## 1. Introduction

Cardiovascular magnetic resonance (CMR) imaging is gaining traction as a preferred imaging modality for comprehensive non-invasive cardiac assessment due to its unparalleled soft tissue contrast in the absence of iodinated contrast and ionizing radiation (1). However, the availability and accessibility of CMR is still limited (2). Whole-body magnetic resonance imaging (MRI) systems have historically evolved toward higher field strength with stronger and faster gradients, primarily driven by the demand for higher signal-to-noise ratio (SNR), imaging speed, and spatial resolution. While higher field strength and faster gradients have afforded significant advantages such as higher spatial and temporal resolution at reduced scan time, these come at the cost of systems that are more expensive to manufacture, install, and maintain. Balancing the technical and economic aspects of an MRI system can therefore impose constraints on its utility. For example, the bore dimensions of higher field systems (60–70 cm) limit the range of body habitus that can be accommodated in the scanner. Economic limitations associated with the manufacture and siting costs of high-field MRI systems constrain access to MRI scanners globally. Manufacturing costs can be reduced by reducing the main magnetic field strength, and limiting gradient performance. Innovations in superconducting magnet cooling technology can significantly reduce the amount of helium required per MR systems regardless of field strength and further contributes to cost reduction. Lower cost, low-field MRI systems incorporating such advances could address some of these economic issues limiting global access (3, 4), but is comprehensive CMR feasible on a system with reduced field strength and gradient performance, with correspondingly lower SNR and slower imaging speed?

The feasibility of contemporary CMR techniques has been explored (5) and demonstrated (6–9) in recent years on low-field (B0) systems. The image quality and the array of cardiac applications demonstrated in these recent studies have already surpassed expectations based on the relatively poor performance of previous generations of low-field systems. The low-field systems used in these recent publications were either ramped down commercially available 1.5 T systems (7, 9) or hybrid MRI—radiation therapy systems (5, 6, 8) for image-guided radiotherapy; in both cases these included high performance gradient systems. A commercial whole body 0.55 T MRI system (MAGNETOM Free.Max, Siemens Healthcare, Erlangen, Germany) has recently become available with an ultra-wide 80 cm bore. This contemporary system possesses several innovations in hardware design, with minimal usage of helium, and lighter weight for greater portability and flexibility in siting. One significant limitation for cardiac imaging, however, is the gradient system which has a maximum amplitude of 26 mT/m and slew rate of 45 T/m/s. In comparison, 1.5 T and 3 T systems typically used for cardiac imaging have gradient specifications of 45 mT/m and 200 T/m/s. It is also important to note that this difference in gradient performance distinguishes the work presented here from recent publications demonstrating CMR on prototype scanners with high performance gradient systems ramped down to 0.55 T (7, 9). The most direct impact of these comparatively slower and lower amplitude gradients is longer repetition (TR) and echo times (TE), and hence, longer imaging times, and/or reduced spatial and temporal resolutions.

Under these conditions of low-field strength and gradient performance, we sought to implement the cardiac techniques needed for a comprehensive cardiac MRI exam on this commercially available system. To overcome the challenges of low SNR and limited gradient performance, we leveraged the use of compressed sensing (CS) (10) based image acquisition and reconstruction methods to boost SNR and support the higher acceleration rates needed to overcome the gradient constraints while maintaining reasonable scan times. We sought to maintain the Society of Cardiovascular Magnetic Resonance (SCMR) recommended guidelines for clinical CMR protocols (11). The successes, challenges, and lessons learned in these initial efforts to build a comprehensive CMR exam are described in this work, and example images are shown in porcine infarct models, healthy volunteers, and patients with cardiovascular disease. While further optimization efforts are warranted to ensure robust implementation of the cardiac applications described here prior to clinical utilization, and validation requires careful objective assessments including comparisons to clinically standard higher field strength systems, the goal of this pilot work is to demonstrate the feasibility of CMR on this novel 0.55 T system. Accordingly, each section below is structured such that a specific CMR technique is described including the implementation details and the corresponding imaging protocol in its current state of optimization, together with resulting exemplary images.

## 2. MRI system and subject preparation

All animal and human subject studies were performed on a commercial 0.55 T MR system (MAGNETOM Free.Max, Siemens Healthineers, Erlangen, Germany). The system has a bore size of 80 cm diameter with a table weight limit of 550 lbs. The system gradients have a maximum amplitude of 26 mT/m and slew rate of 45 T/m/s. All animal studies were approved by the Institutional Animal Care and Use Committee. The animals were anesthetized with isoflurane and mechanically ventilated on 100% oxygen during the study. Breath-hold for cardiac sequences was achieved by holding the ventilator at expiration. All human subject studies were approved by The Ohio State University Biomedical Sciences Institutional Review Board and participants provided written informed consent. As the system is not equipped with an electrocardiographic (ECG) monitoring and triggering unit, two different MRI compatible patient monitoring devices (3880 MRI Patient Monitoring System, IRADIMED, Winter Springs, FL; and Expression MR400, Philips N.V., Amsterdam, The Netherlands) were used to provide external trigger inputs to the system. All participants and animals were positioned head-first into the scanner bore. Following ECG electrode placement, a large flexible six-element phased array receiver coil (two rows of three elements each) was placed over the chest and used in combination with the nine spine coil elements (three rows of three elements each) embedded within the magnet bore and under the patient table. All cardiac imaging sequences investigated in this study are not commercially available and are prototype research pulse sequences that were either provided by the manufacturer under our research collaboration, or developed in-house at our institution.

## 3. Cine for biventricular structure and function

**Figure 1:**
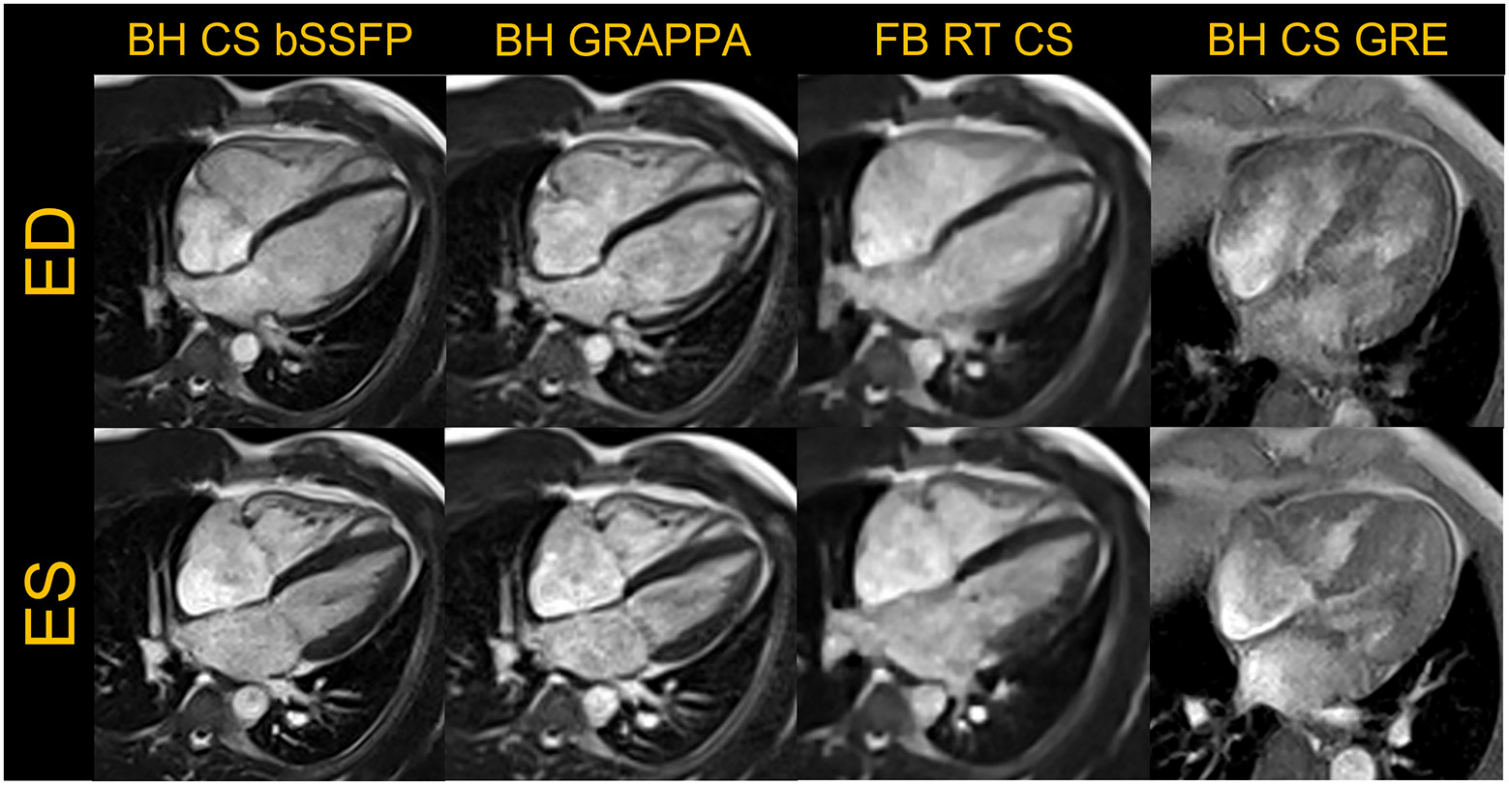
Representative examples of images acquired in a four-chamber view are shown in the end-diastole (ED) and end-systole (ES) frames for breath-held (BH) compressed sensing (CS) and GRAPPA based segmented bSSFP cine, free-breathing (FB) real-time (RT) CS bSSFP cine and BH CS based GRE cine are shown. The GRE images were acquired in a different volunteer.

We applied both balanced steady-state free precession (bSSFP) and gradient echo (GRE) cine, and also implemented breath-held segmented and free-breathing real-time (RT) cine on the 0.55 T system (Figure 1). Acquisition parameters for breath-hold segmented and RT cine are listed in Table 1 along with cine parameters used on our institution's 1.5 T system (MAGNETOM Sola, Siemens Healthcare, Erlangen, Germany) for comparison.

**Table 1:** Cine acquisition parameters for 0.55 T in comparison to 1.5 T.

	**BH-cine**		**RT-cine**	
Scanner	1.5 T	0.55 T	1.5 T	0.55 T
Flip angle (°)	80	110	80	110
TE (ms)	1.16	1.95	1.04	1.84
TR (ms)	2.71	4.65	2.43	4.55
RBW (Hz/pixel)	930	930	1,184	1,002
Lines per HB	12	6	18	10
Temporal resolution (ms)	32.5	27.9	43.7	45.5
Slice thickness (mm)	6	8	8	8
Pixel size (mm)	1.8 × 1.8	1.8 × 1.8	2.0 × 2.0	1.8 × 1.8
CS acceleration rate	4.3	4.3	7.4	13
Scan time (HB)	3	6	1	1

BH, breath-held; RT, real-time; TE, echo time; TR, repetition time; RBW, receiver band width; CS, compressed sensing; HB, heartbeats.

**Figure 2:**
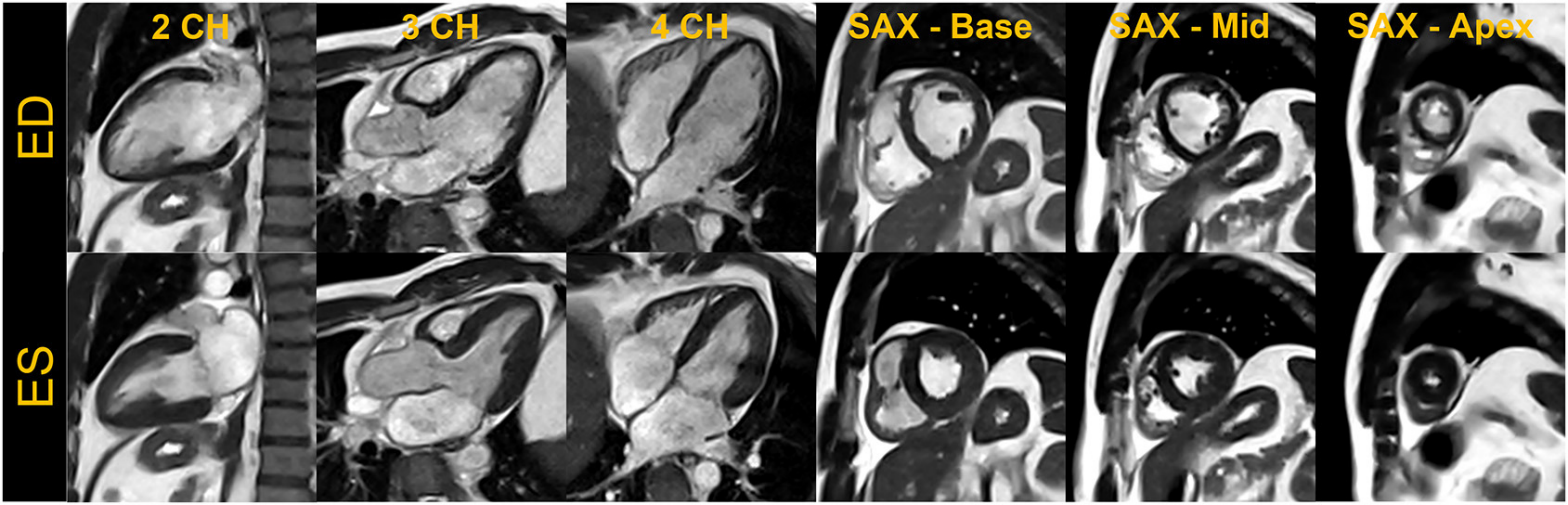
End-diastole (ED) and end-systole (ES) cardiac frames from a compressed sensing-based breath-held segmented balanced steady-state free precession cine. Images acquired from a single volunteer are shown in standard cardiac views– two-chamber (2CH), three-chamber (3CH), four-chamber (4CH) and base, mid, and apical slices in short-axis (SAX).

**Figure 3:**
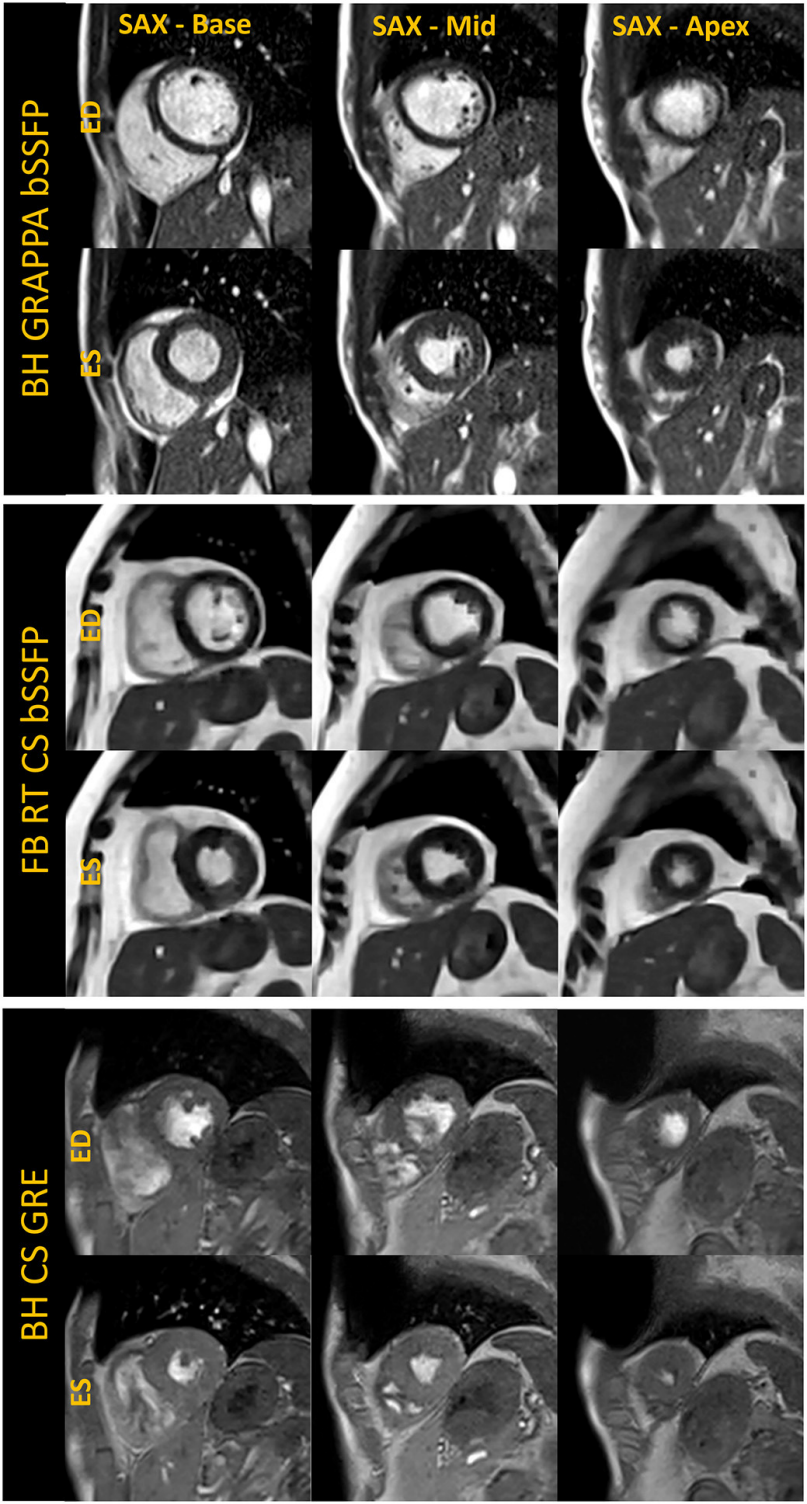
Basal, mid, and apical short-axis cine images are shown in the end-diastole (ED) and end-systole (ES) cardiac frames for breath-held segmented GRAPPA bSSFP, free-breathing real-time CS bSSFP and breath-held segmented CS GRE method. The images shown for each technique were acquired in separate volunteers.

For breath-held segmented bSSFP cine, a CS-accelerated sequence with a variable density sampling k-space pattern and Cartesian readout was utilized. Figure 2 shows representative end-diastole and end-systole images from a volunteer in standard cardiac views. The acquired spatial resolution was set to match protocols on our clinical 1.5 T and 3 T systems (MAGNETOM Vida, Siemens Healthcare, Erlangen, Germany). The acceleration rate was adapted based on the subject's R-R interval (a typical rate was 4.3) to maintain a temporal resolution of ~30 ms, meeting recommendations of the SCMR (11). The number of iterations and the regularization parameters for the CS reconstruction were determined based on informal visual perception of image quality, signal-to-noise, and artifact. Asymmetric sampling was utilized to minimize TE and TR for the cine; however, limited gradient slew rate prolonged the TE and TR on the 0.55 T system when compared to 1.5 T. Specific Absorption Rate (SAR) scales with the square of the field strength; therefore, higher flip angles of 90° to 110° were possible for bSSFP cine at 0.55 T (8) as compared to 1.5 T or 3 T, where maximum flip angles are in the range of 50° (3 T) to 80° (1.5 T). A flip angle in the range of 110° has previously been shown to maximize blood-myocardium contrast in bSSFP (8). While our clinical protocols at 1.5 T and 3 T using the same sequence are set to a three-shot acquisition requiring three heartbeats (HB) per slice, at 0.55 T, the acquisition time was increased to six heart beats per slice to meet spatial and temporal resolution requirements, without pushing the acceleration rate too far. On the other hand, a segmented GeneRalized Autocalibrating Partial Parallel Acquisition (GRAPPA) bSSFP cine with rate 2 acceleration, 24 separate reference lines, and similar acquisition parameters required 10 HB per slice at 0.55 T (Figures 1, 3) when compared to the CS implementation.

For free-breathing real-time cine, a CS-accelerated bSSFP sequence was implemented with an acceleration rate 13, similar to what is used at higher field with an acquisition time of 2.5 seconds per slice (Figures 1, 3). The first complete HB was then reconstructed as a separate series with a consistent number of frames to facilitate quantification. Other image acquisition parameters were matched to the segmented bSSFP cine at 0.55 T, with 10 lines acquired per HB leading to a temporal resolution of 45.5 ms. The segmented cine sequences were reconstructed on the scanner using a spatial temporal L1 regularization with wavelet transform, while the RT data were reconstructed inline using the Gadgetron-based (12) implementation of SCoRe (Sparsity adaptive Composite Recovery) (13), which is a CS-based parameter-free reconstruction method. SCoRe can utilize multiple sparsifying transforms while providing data-driven adjustment of the regularization weights. Both non-decimated wavelet and temporal PCA were utilized as sparsifying transforms to capture the local and non-local structure. Commercially standard spatial interpolation and image filters available from the manufacturer were enabled for both segmented and RT scans to enhance image sharpness.

While the reduced SNR at low field can make spoiled gradient echo (GRE) based sequences challenging, GRE is often used in patients with implanted devices to limit metal artifacts; therefore, we implemented a segmented CS GRE cine sequence using TR/TE 7.02/2.99 ms, flip angles around 15–25 degrees and receiver bandwidth (RBW) of 250 Hz/pixel. Images acquired in a healthy volunteer are shown in Figures 1, 3. Other acquisition parameters were similar to the segmented CS bSSFP sequence. Although blood-myocardium contrast appears to be lower than at higher B0 upon visual inspection, these initial images are encouraging and warrant additional investigation to evaluate the utility of GRE cine in patients with implanted devices.

As the exemplary images in Figures 1–3 depict, it appears that the overall image quality of these cine methods will be sufficient to facilitate quantitative evaluation, although this will require additional detailed analysis. The limitations we experienced include the presence of ringing artifact in some of the cine images, possibly from the longer TR. In addition for the CS cine, the lower SNR of the underlying data, and higher acceleration required may have contributed to the need to use comparatively stronger regularization with respect to our 1.5 T and 3 T protocols; this in turn may have led to the patch-like artifacts in the CS based cine images shown in Figures 1–3. The right ventricle myocardium appears grayer with respect to the left ventricle; this could be due to partial volume with the blood pool and blurring caused by the spatial and temporal regularization used in the CS reconstruction. Further assessments and evaluations are required to confirm and address these challenges.

## 4. Blood flow quantification

**Figure 4:**
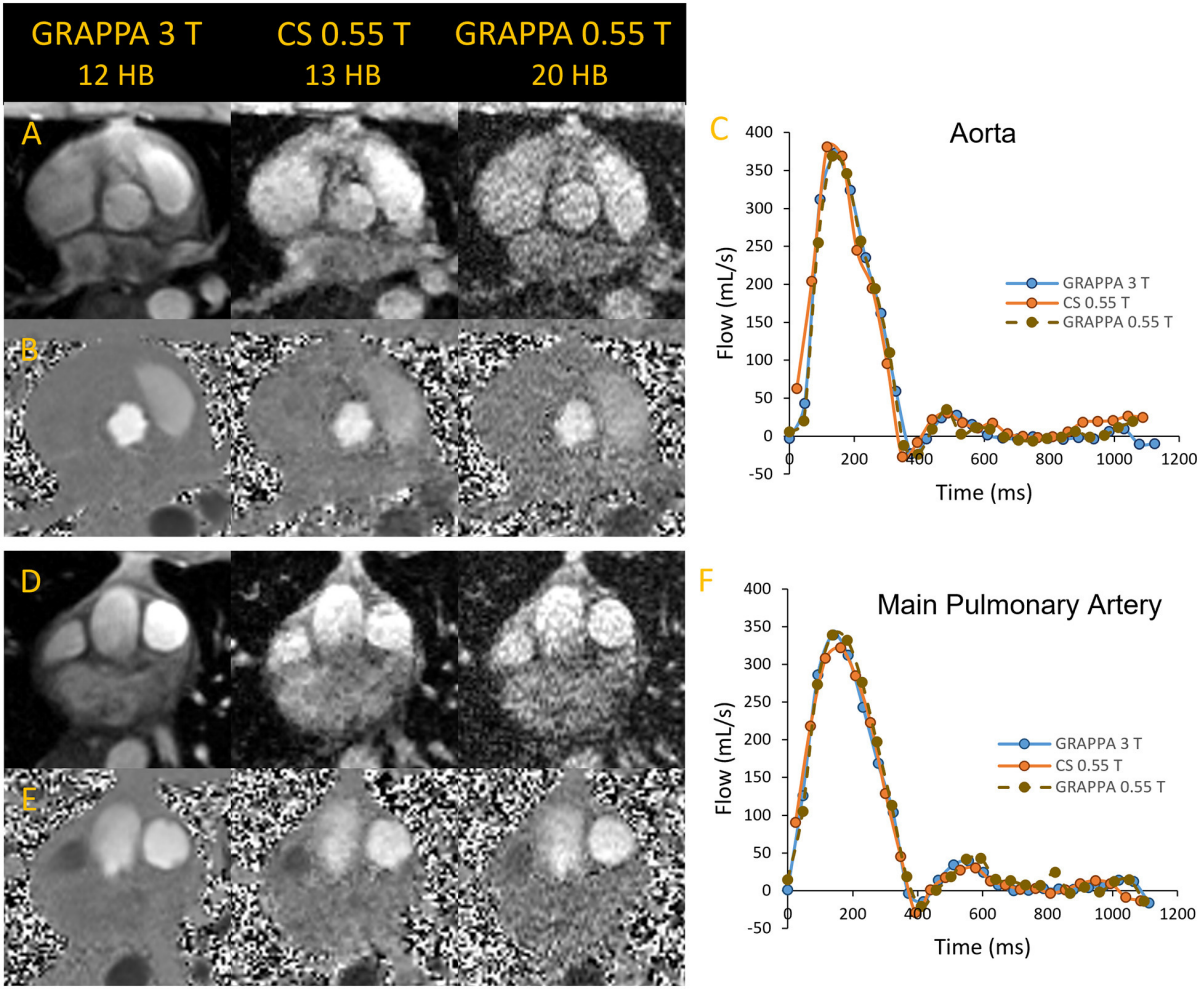
Magnitude and phase images of the aorta **(A, B)** and main pulmonary artey **(D, E)** acquired in a volunteer using segmented GRAPPA at 3 T as reference and compared to compressed sensing and GRAPPA images at 0.55 T. The flow curves corresponding to the images are shown in **(C, F)**.

Phase contrast (PC) imaging is the standard MRI method used to measure blood flow (14). While low-field may offer the advantages of reduced susceptibility and greater homogeneity, the inherently low SNR of the GRE sequences used for PC MRI can be challenging at low-field, and higher acceleration is needed to overcome slower gradients. We implemented breath-held, segmented CS and GRAPPA-based 2D PC MRI methods on this 0.55 T system, matching the spatial and temporal resolution to the standard GRAPPA sequence used at our 3 T system. The acquisition parameters are listed in Table 2 and representative images from a volunteer are shown in Figure 4. To account for the longer TR due to limited gradient performance, the number of lines per HB at 0.55 T was reduced to match temporal resolution, and the acceleration rate of the CS PC MRI sequence was adjusted to approximately match the scan time of the GRAPPA sequence at 3 T.

**Table 2:** Acquisition parameters of phase contrast MR images for 0.55 T in comparison to 3 T.

	**GRAPPA at 3 T**	**CS at 0.55 T**	**GRAPPA at 0.55 T**
TR (ms)	4.43	7.41	7.41
TE (ms)	2.43	4.26	4.26
RBW (Hz/pixel)	501	427	427
FA (°)	10	12	12
Slice thickness (mm)	6	6	6
FOV (mm^2^)	(320–377) × (240–282)	(320–377) × (240–282)	(320–377) × (240–282)
Matrix size	192 × 154	192 × 154	192 × 154
Temporal resolution (ms)	44.3	44.5	44.5
Acquisition time (HB)	12	13	20
Acceleration rate	2	3	2
Lines per HB	5	3	3

GRAPPA, GeneRalized Autocalibrating Partial Parallel Acquisition; CS, compressed sensing; TE, echo time; TR, repetition time; RBW, receiver band width; FA, flip angle; FOV, field of view; HB, heartbeats.

We found that GRAPPA PC MRI at 0.55 T required 20 HB, while the scan time of CS PC MRI at 13 HB was comparable to 3 T GRAPPA PC MRI at 12 HB. As shown in Figure 4, the magnitude and phase images at 0.55 T appear noisier than the corresponding 3 T images; however, the flow quantification results in this example were comparable between the two systems. Stronger regularization parameters than generally applied on higher B0 systems were implemented for the CS PC MR images to achieve the image quality demonstrated in Figure 4. The CS images shown appear to have higher SNR than the corresponding GRAPPA images at 0.55 T. Thus, the CS PC MRI sequence could have an advantage over GRAPPA at 0.55 T, as it provides the acceleration needed to nominally match the spatial and temporal resolution, and scan time of our standard GRAPPA PC MRI sequence at higher B0. Studies are ongoing to validate the accuracy of flow quantification in volunteers and patients with flow defects with respect to higher B0 systems.

## 5. Tissue characterization

### 5.1. Dark-blood sequences

**Figure 5:**
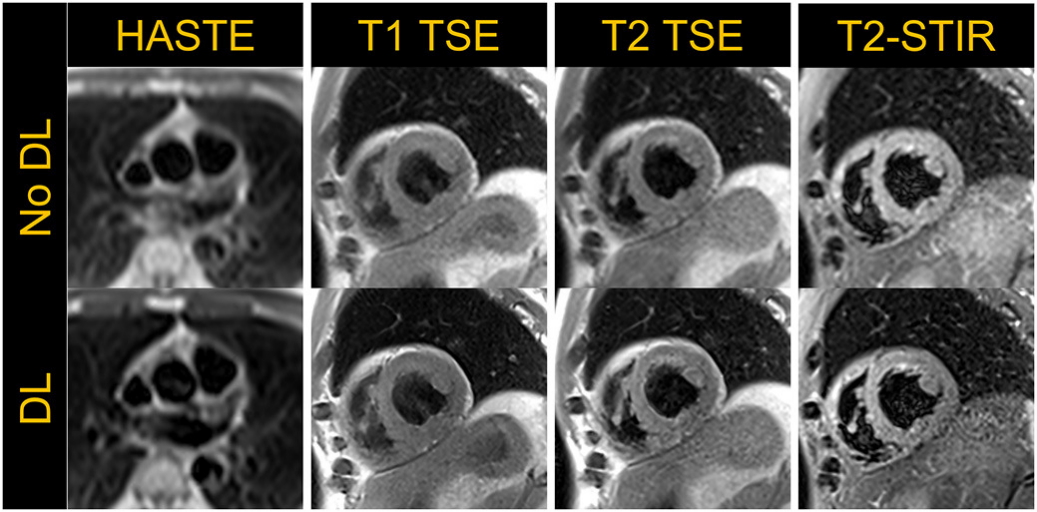
Half-Fourier Acquisition Single-Shot Turbo Spin Echo (HASTE) in an axial view and T1-weighted, T2-weighted TSE and T2-short tau inversion recovery (STIR) images acquired in a mid-short axis view in a volunteer demonstrate the application of dark-blood turbo spin echo based cardiac imaging techniques. The **top row** shows images without deep-learning (DL) based image enhancement while the **bottom row** depicts images with increased sharpness resulting from DL-based image reconstruction.

Although quantitative parametric mapping techniques are replacing qualitative T1 and T2 weighted dark-blood turbo spin echo (TSE) sequences for myocardial tissue characterization, TSE sequences remain useful to distinguish morphological features and to characterize masses and tumors. We therefore sought to implement dark-blood Half-Fourier Acquisition Single-shot Turbo Spin Echo (HASTE), as well as T1 and T2 weighted, and T2 weighted short-tau inversion recovery (T2-STIR) TSE sequences (15, 16). Image acquisition parameters for these three sequences are listed in Table 3, and example images are shown in Figure 5. Deep Resolve Gain, an iterative denoising method, and Deep Resolve Sharp, an artificial intelligence-based image reconstruction method to increase image sharpness available from the manufacturer for TSE sequences, were used for targeted denoising to improve SNR, and to enhance image sharpness and resolution as demonstrated in Figure 5. Given that TSE images tend to have relatively high SNR, and the sequence is not as reliant on fast gradients as other techniques, it is not surprising that TSE performs well at low-field with little modification. Although evaluation of these sequences in individuals with known pathology is yet to be performed to demonstrate clinical utility, these preliminary examples in healthy volunteers are encouraging. The images also show the potential for deep-learning-based enhancement, which was utilized in the TSE images shown in this work.

**Table 3:** Image acquisition parameters for dark-blood cardiac sequences.

**Sequence**	**FA (°)**	**TE (ms)**	**Echo spacing (ms)**	**RBW (Hz/pixel)**	**Turbo factor**	**Acquisition window (ms)**	**Slice thickness (mm)**	**Pixel size (mm^2^)**	**Acceleration**	**Scan time (HB)**
T1-TSE	180	33	5.46	401	11	63	6	1.8 × 1.8	G 2	10
T2-TSE	180	44	5.46	401	14	75	6	2.0 × 2.0	G 2	12
T2-STIR	180	43	6.18	362	20	127	6	2.0 × 2.0	G 2	15
HASTE	160	16	4.1	1,502	72	295	10	3.6 × 3.6	G 2	1

TSE, turbo spin echo; STIR, short-tau inversion recovery; HASTE, Half-Fourier Acquisition Single-shot Turbo Spin Echo; BH, breath-held; RT, real-time; CS, compressed sensing; GRAPPA, GeneRalized Autocalibrating Partial Parallel Acquisition; TE, echo time; TR, repetition time; RBW, receiver band width; FA, flip angle; FOV, field of view; HB, heartbeats.

### 5.2. Myocardial parametric mapping

Myocardial longitudinal (T1) and transverse (T2) relaxation times are elevated with fibrosis, edema, and inflammation. Quantitative myocardial parameter mapping methods are being used clinically at higher field to evaluate myocardial tissue characteristics (17, 18). We implemented parameter mapping schemes for T1 and T2 taking into consideration the shorter T1 relaxation times and longer T2 relaxation times at 0.55 T in comparison to higher field. The image acquisition parameters are listed in Table 4. The primary modification at 0.55 T we have currently implemented is an increase in the number of source images; this is expected to boost SNR of the resulting parametric maps through the pixel-wise parameter fitting process.

**Table 4:** Acquisition parameters for T1 and T2 mapping sequences at 0.55 T in comparison to 1.5 T.

	**T1 mapping**		**T2 mapping**	
Scanner	1.5 T	0.55 T	1.5 T	0.55 T
FA (°)	35	50	70	70
TE (ms)	1.01	1.77	1.04	1.69
TR (ms)	2.42	4.3	2.43	4.18
RBW (Hz/pixel)	1,085	539	1,184	558
Lines per HB	60	60	55	60
Temporal resolution (ms)	145	258	133	250
Slice thickness (mm)	8	10	8	10
Pixel size (mm)	2.0 × 2.0	2.4 × 2.4	2.1 × 2.1	2.4 × 2.4
Acceleration	G 2	G 2	G 2	G 2
Scan time (HB)	11	14	7	16

BH, breath-held; RT, real-time; CS, compressed sensing; GRAPPA, GeneRalized Autocalibrating Partial Parallel Acquisition; TE, echo time; TR, repetition time; RBW, receiver band width; FA, flip angle; FOV, field of view; HB, heartbeats.

As the native myocardial T1 time is shorter at 0.55 T [~700 ms (7)], additional data samples at shorter inversion times are desirable in comparison to the standard data acquisition schemes at higher B0. Therefore, we implemented a single-shot inversion recovery prepared bSSFP readout modified Look-Locker inversion recovery (MOLLI) based T1 mapping method with a 4(1)3(1)2(1)2 acquisition strategy, using four inversion pulses to collect 11 source images within 14 HB. The flip angle was set higher at 50° with the expectation of improved SNR. The same acquisition scheme was utilized for both pre- and post-contrast T1 mapping.

For T2 mapping, a total of 6 single-shot T2 prepared bSSFP images were acquired with three T2 preparation times of 0, 25, and 60 ms, repeated twice with two recovery beats in between each image, resulting in a total acquisition time of 16 HB.

A thicker 10 mm slice was used to increase SNR in both T1 and T2 mapping. GRAPPA parallel imaging was employed with acceleration rate 2 and 60 separate reference lines. Cartesian trajectory with linear k-space reordering was used for both T1 and T2 maps. Non-rigid inline motion correction available from the manufacturer was used to register the source images prior to pixel-wise estimation of relaxation time. All source images were co-registered, and for T2 mapping, images acquired at the same preparation time were not explicitly averaged but contributed to the curve fitting process.

**Figure 6:**
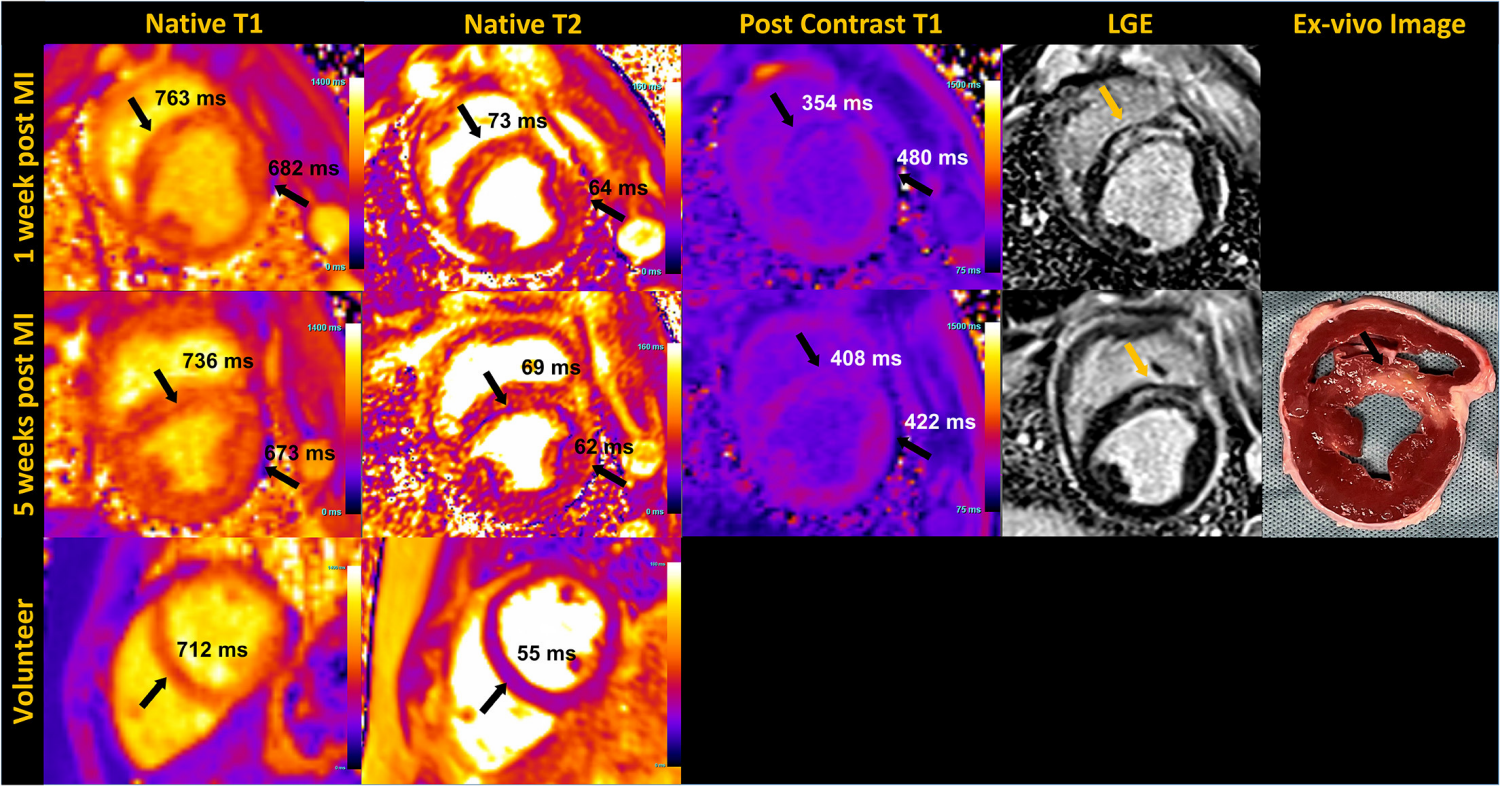
Mid short-axis quantitative myocardial native T1, T2 map, post contrast T1 map and phase sensitive inversion recovery late gadolinium enhanced (LGE) images in an animal at 1 week and 5 weeks post-myocardial infarction (MI) are shown in the **top two rows**. The arrows pointing to the septum on T1 and T2 maps indicate elevated native T1 and T2, and reduced post-contrast T1 corresponding to infarct location, confirmed by LGE and the unstained *ex-vivo* image. T1 and T2 values of the remote myocardium in the lateral region are also indicated. Sub-endocardial rim artifact are seen on the maps due to the animal's high heart rate. The **bottom row** shows native T1 and T2 maps acquired in a healthy volunteer in the mid-short axis view.

The application of T1 and T2 mapping sequences were investigated in both healthy volunteers and a porcine infarct model (Figure 6). Post-contrast T1 was shorter and native T2 was higher in infarct regions compared to remote myocardium during the acute stage post-MI in the animal images shown. As prolonged breath-hold durations could be tolerated in the animal studies, we incorporated additional source images into the T1 (30 images, 60 HB) and T2 (12 images, 45 HB) mapping protocols, with the intention to increase the SNR through effective averaging. Such extended acquisition times would only be feasible in human subjects during free-breathing while employing an effective strategy for respiratory motion compensation.

Our preliminary assessment of T1 and T2 mapping at 0.55 T demonstrates feasibility, but still requires considerable investigation for clinical translation. The extended breath-hold of 14 to 16 HB we implemented for the volunteers may be too long for cardiac patients. Ringing artifacts resulting from the poor temporal resolution (~250 ms) were observed in some T2 maps, as shown in the porcine images. In some instances, these artifacts extended across the septum, resulting in lower T2 measured in the septum compared to the lateral wall. In addition, the motion correction algorithm used routinely with success at higher field has been challenging at 0.55 T, most likely due to the low SNR of the individual source images. In instances where the motion correction did not perform well, deformation of the individual motion-corrected images resulted in blurring of the myocardial boundaries in the resulting maps. Further research into optimal image registration and motion correction strategies are ongoing.

## 6. First-pass perfusion

First-pass perfusion imaging is limited in SNR and relies on fast gradients, making it an especially challenging application on a low-field system with limited gradient performance. A prototype first-pass perfusion sequence using CS reconstruction to provide sufficient acceleration was investigated in a porcine infarct model and applied in healthy human volunteers at 0.55 T under resting conditions. A 0.075 mmol/kg dose of gadobutrol (Gadavist, Bayer Healthcare, Whippany, NJ) was administered to the animals and volunteers at 4 ml/second. The data were collected during free-breathing using a fat-suppressed, saturation-recovery T1-weighted GRE sequence. The scan parameters were: FOV ~380 × 296 mm^2^, TE/TR 1.81/4.25 ms, image matrix 160 × 125, flip angle 90°, acceleration rate 5 using pseudo-random variable density sampling, called Golden Ratio Offset Sampling (GRO) (1), temporal footprint 90–108 ms, spatial resolution 2.0–2.38 × 2.0–2.38 mm^2^, and 60 repetitions (cardiac cycles). The acquired data were reconstructed inline using a Gadgetron (2) implementation of SCoRe (3). All the repetitions were averaged to generate the fully sampled autocalibration region, and an eigenvalue approach to autocalibrating (ESPIRiT) method (4) was used to extract the coil sensitivity maps. To capture the local and non-local structures, both non-decimated wavelet (local) and temporal principal component analysis (non-local) were utilized as sparsifying transforms. The reconstruction time is ~5 s per anatomical slice acquired (including all 60 repetitions) using our GPU workstation (NVIDIA GTX 3090) (12, 13, 19, 20).

**Figure 7:**
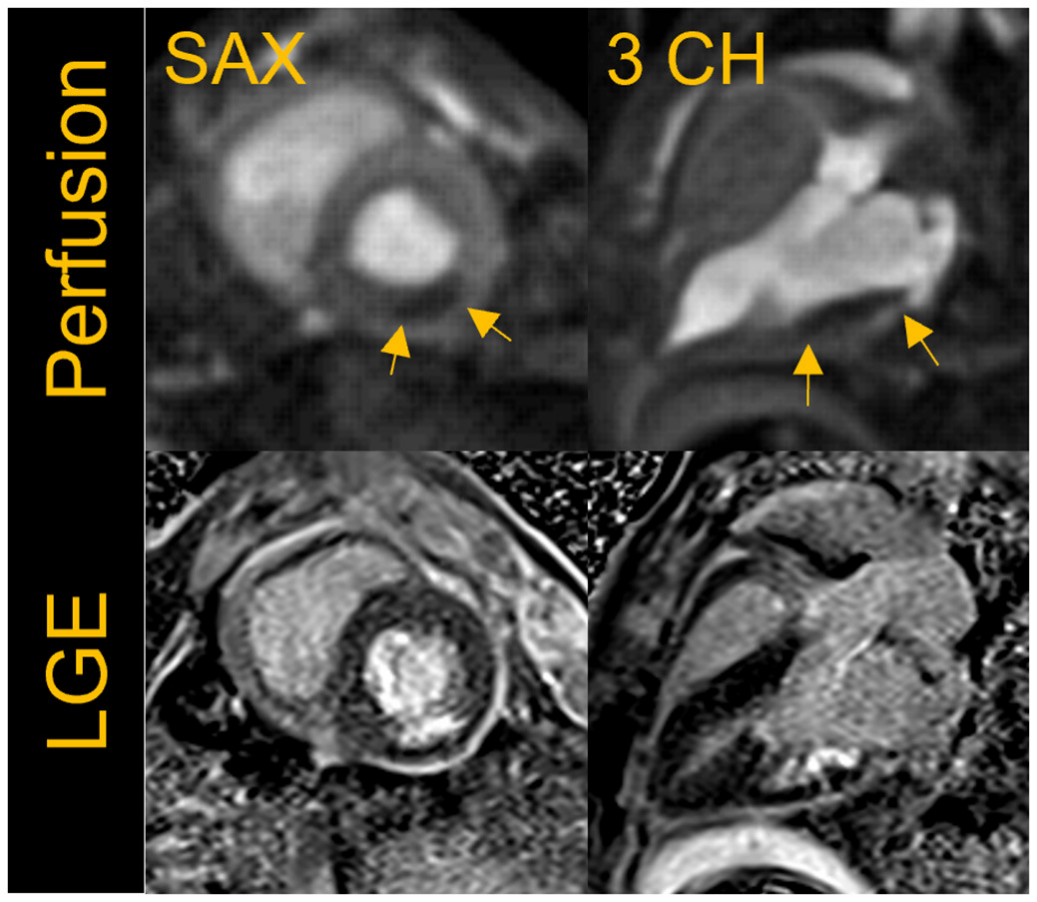
First-pass perfusion images acquired at rest with a fat suppressed, saturation-recovery T1-weighted gradient echo (GRE) sequence shows rest perfusion defect in the short-axis (SAX) and three- chamber view in a porcine infarct model where the left circumflex artery was occluded. The images shown here were acquired ~5 weeks post-myocardial infarction (MI). The perfusion defect visually correlates well to the region of infarct as seen in the corresponding breath-held segmented late gadolinium enhancement (LGE) images.

Figure 7 shows an example of resting perfusion images indicating a perfusion defect in a region of known myocardial infarction. The perfusion images acquired in healthy volunteers were determined to have visually comparable image quality to that of the animal studies. Further work is needed to quantitatively assess the perfusion defects and to validate the technique across different B0 systems. The results shown were acquired using state-of-the art image reconstruction technology developed in-house, and fast computer hardware to achieve highly accelerated real-time cine and perfusion imaging with rapid, in-line reconstruction. While such technology is not widely available currently, our preliminary results represent a promising pathway toward global realization of these CMR techniques.

## 7. Late gadolinium enhancement (LGE) imaging

**Figure 8:**
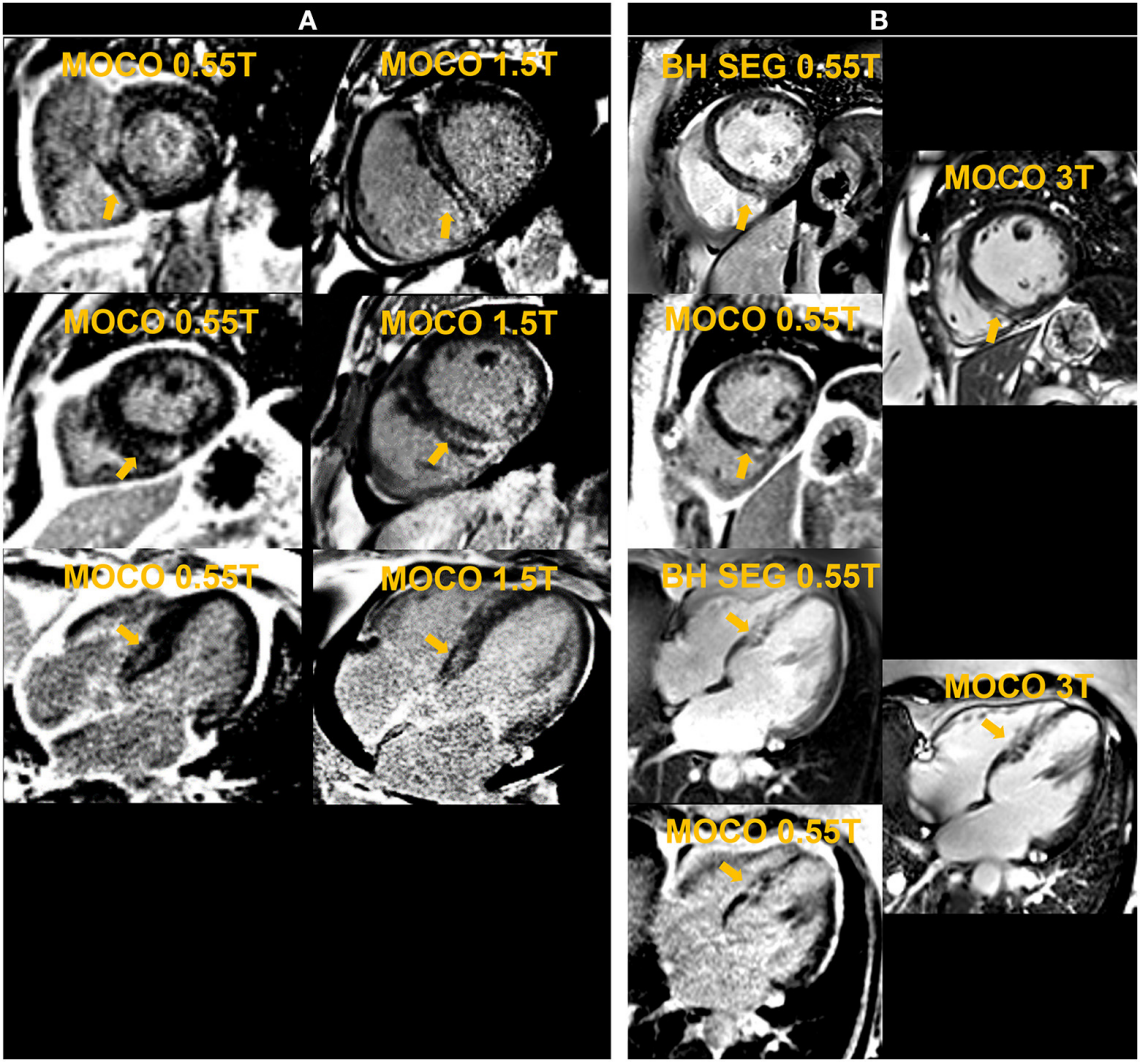
The left panel shows motion-corrected and averaged free-breathing single-shot late gadolinium enhancement images (MOCO LGE) in a patient [Patient **(A)**] demonstrating mid-wall non-ischemic fibrosis pattern at 0.55 T and 1.5 T. The right panel shows breath-held (BH) segmented (SEG) and MOCO LGE images acquired at 0.55 T in comparison to corresponding MOCO LGE images at 3 T in another individual [Patient **(B)**] and demonstrates infarct scar at the inferior septum.

Late gadolinium enhancement (LGE) reveals myocardial scar and fibrosis and is a key component of the basic CMR exam. The standard technique is based on inversion recovery (IR) prepared GRE or bSSFP (21) and tends to have lower SNR than other techniques and therefore may be challenging at low-field (22). We implemented and evaluated both breath-held segmented and motion-corrected and averaged free-breathing (MOCO) single-shot IR-prepared bSSFP sequences for LGE imaging (23); the acquisition parameters are listed in Table 5. LGE images were typically acquired from 8 to 10 min post-contrast injection. A total dose of 0.15 mmol/kg of gadobutrol (Gadavist, Bayer Healthcare, Whippany, NJ) was administered to generate the animal and human images shown in Figures 6–8, demonstrating examples of positive LGE findings.

**Table 5:** Acquisition parameters for late gadolinium enhancement (LGE) images at 0.55 T in comparison to 1.5 T.

	**BH LGE**		**MOCO LGE**	
Scanner	1.5 T (GRE)	0.55 T (SSFP)	1.5 T	0.55 T
FA (°)	20	80	55	50
TE (ms)	1.55	2.48	1.18	1.84
TR (ms)	4.06	6.66	2.79	4.69
RBW (Hz/pixel)	465	200	1,085	698
Lines per HB	31	21	86	63
Temporal resolution (ms)	142	140	240	295
Slice thickness (mm)	8	10	8	8
Pixel size (mm)	1.4 × 1.4	1.6 × 1.6	1.4 × 1.4	1.5 × 1.5
Acceleration	None	None	G 2	G 2
Scan time (HB)	8	12	16	24

LGE, late gadolinium enhanced; BH, breath-held; MOCO, free breathing motion corrected and averaged; RT, real-time; CS, compressed sensing; GRAPPA, GeneRalized Autocalibrating Partial Parallel Acquisition; TE, echo time; TR, repetition time; RBW, receiver band width; FA, flip angle; FOV, field of view; HB, heartbeats.

For free-breathing LGE, twelve averages requiring 24 HB per slice were used to boost SNR, compared to the standard 8 averages employed in our 1.5 T and 3 T protocols. We observed failures of the motion correction algorithm, similar to what we observed in cardiac mapping, most likely due to the low SNR of the single-shot source images. This limited the benefits of increased averaging. The preliminary example images from animals and patients with positive LGE confirmed on 1.5 T and 3 T systems are promising. Additional assessments are warranted to correlate the extent of LGE to that depicted by 1.5 T and 3 T systems.

## 8. Angiography

MR angiography, with and without gadolinium-based contrast agents, is an important component of the comprehensive cardiovascular imaging exam. Thoracic MRA is widely used in the evaluation of aortic disease, congenital heart disease, and to map out the anatomy of the pulmonary veins. We implemented magnetization-prepared non-contrast MRA and contrast-enhanced MRA on the Free.Max system. Acquisition parameters for all protocols are listed in Table 6.

**Table 6:** Acquisition parameters for magnetic resonance angiogram (MRA) images at 0.55 T in comparison to 1.5 T.

	**Non-contrast MRA bSSFP**		**Gated contrast MRA GRE**	
Scanner	1.5 T	0.55 T	1.5 T	0.55 T
FA (°)	90	110	30	30
TE (ms)	1.45	1.89	1.25	1.71
TR (ms)	3.38	4.61	2.97	3.68
RBW (Hz/pixel)	592	501	591	781
Lines per HB	35	35	100	80
Temporal resolution (ms)	118	161	297	294
Slice thickness (mm)	1.3	1.5	1.4	1.5
Pixel size (mm)	1.6 × 1.6	1.6 × 1.6	1.4 × 1.4	1.8 × 1.8
Acceleration	G 2	G 2	CS 9	CS 7
Scan time (HB)	78	142	10	13

MRA, magnetic resonance angiogram; bSSFP, balanced steady-state free precession; GRE, gradient echo; BH, breath-held; RT, real-time; CS, compressed sensing; GRAPPA, GeneRalized Autocalibrating Partial Parallel Acquisition; TE, echo time; TR, repetition time; RBW, receiver band width; FA, flip angle; FOV, field of view; HB, heartbeats.

### 8.1. Non-contrast enhanced MRA

**Figure 9:**
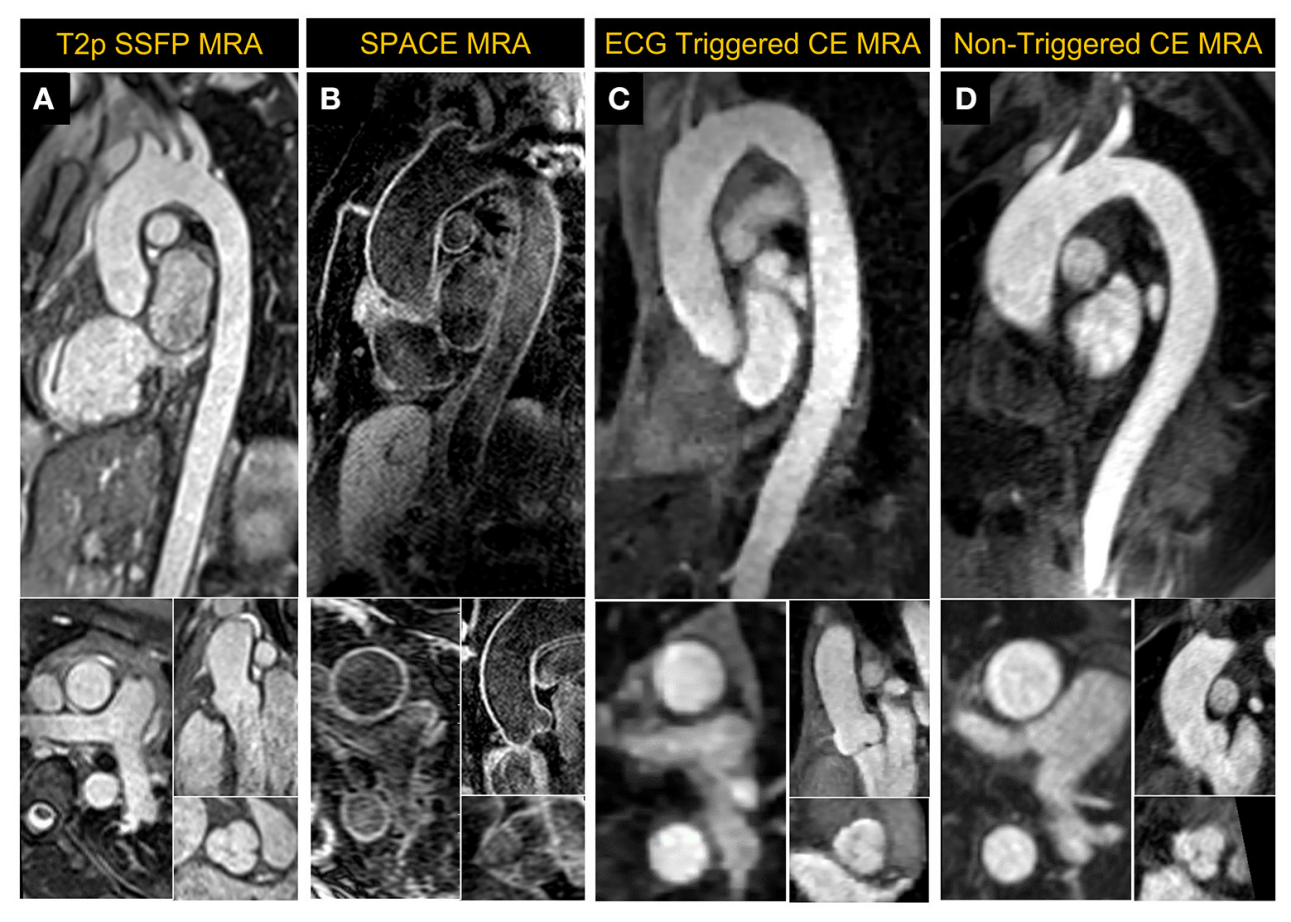
Magnetic Resonance Angiography (MRA) images acquired at 0.55 T. **(A)** 2D reformatted images from ECG-triggered, navigator-gated, GRAPPA-based 3D non-contrast MRA acquired in a healthy volunteer, **(B)** dark-blood 3D SPACE MRA images in a patient with dilated mid ascending aorta and having wires in the thoracic vertebrae post-spinal fusion, **(C)** a compressed sensing-based ECG-triggered contrast enhanced 3D MRA in a healthy volunteer and **(D)** non-gated contrast-enhanced MRA images acquired in a large patient with a mildly dilated thoracic aorta. The patient in **(D)** was unable to fit comfortably and proceed with the exam on a standard scanner but successfully completed a CMR exam on the 0.55 T system.

Non-contrast MRA of the thoracic aorta was implemented using a magnetization-prepared, ECG-triggered, navigator-gated, 3D bSSFP sequence (24). The technique incorporates T2 preparation and fat suppression and is accelerated by a factor of 2 using GRAPPA. The slice thickness was set at 1.5 mm on 0.55 T while 1.3 mm was typically used on our 1.5 T system. An example image acquired in a healthy volunteer at 0.55 T using GRAPPA bSSFP is shown in Figure 9A.

A variable flip-angle, fat-suppressed, dark-blood 3D SPACE MRA (25) sequence was also implemented; example images acquired in a patient with dilated thoracic aorta are shown in Figure 9B. This sequence that is used to provide 3-dimensional dark blood anatomy, being a spin echo approach, is less prone to metal artifact than bSSFP in patients with implanted devices. The acquisition parameters were: TR/TE 4.06/23 ms, temporal resolution 214 ms, acquired pixel size 1.6 × 1.6 × 1.3 mm^3^, averages 2, GRAPPA acceleration rate 2 with 24 integrated reference lines, turbo factor 25 and echo train duration 110 ms, and RBW of 630 Hz/pixel.

### 8.2. Contrast-enhanced MRA

ECG-gating in CE-MRA can significantly reduce image blurring, improve image quality and vessel sharpness in thoracic vessels compared to non-gated acquisitions (26, 27). Conventional parallel imaging acceleration is generally too slow to achieve sufficient spatial resolution and coverage within a reasonable breath hold for ECG-gated MRA, especially on a low-field system with slower gradients. A CS-accelerated ECG-gated CE-MRA was implemented based on a Cartesian 3D FLASH sequence with a variable-density Poisson disk undersampling pattern. CE-MRA was performed during the injection of contrast media at a rate of 2 ml/s (0.1–0.2 mmol/kg), followed by a 25 ml saline flush at the same rate. The ECG-gated CE-MRA sequence started with the breath-hold command as soon as the contrast reached the left ventricle. Figure 9C shows the reformatted 2D images of the thoracic aorta acquired in a volunteer using CS-accelerated ECG-gated CE-MRA.

A slab-selective, 3D CE-MRA without ECG gating was also implemented as an alternative for patients who are incapable of breath-holding (28). Acquisition parameters were: TR/TE 4.18/1.5 ms, acquired pixel size 1.4 × 1.4 × 1.5 mm^3^, flip angle 30°, rate 2 GRAPPA with integrated 24 reference lines and RBW of 350 Hz/pixel. An example image acquired in a patient with dilated thoracic aorta is shown in Figure 9D.

As shown in the reformatted images in Figure 9, the aortic root could be clearly visualized using all techniques at 0.55 T. Stronger regularization effects may have contributed to the slightly pixelated appearance of the CS based ECG-gated CE MRA sequence in comparison to other MRA techniques that were applied.

## 9. Demonstrating clinical utility—Anecdotal evidence

**Figure 10:**
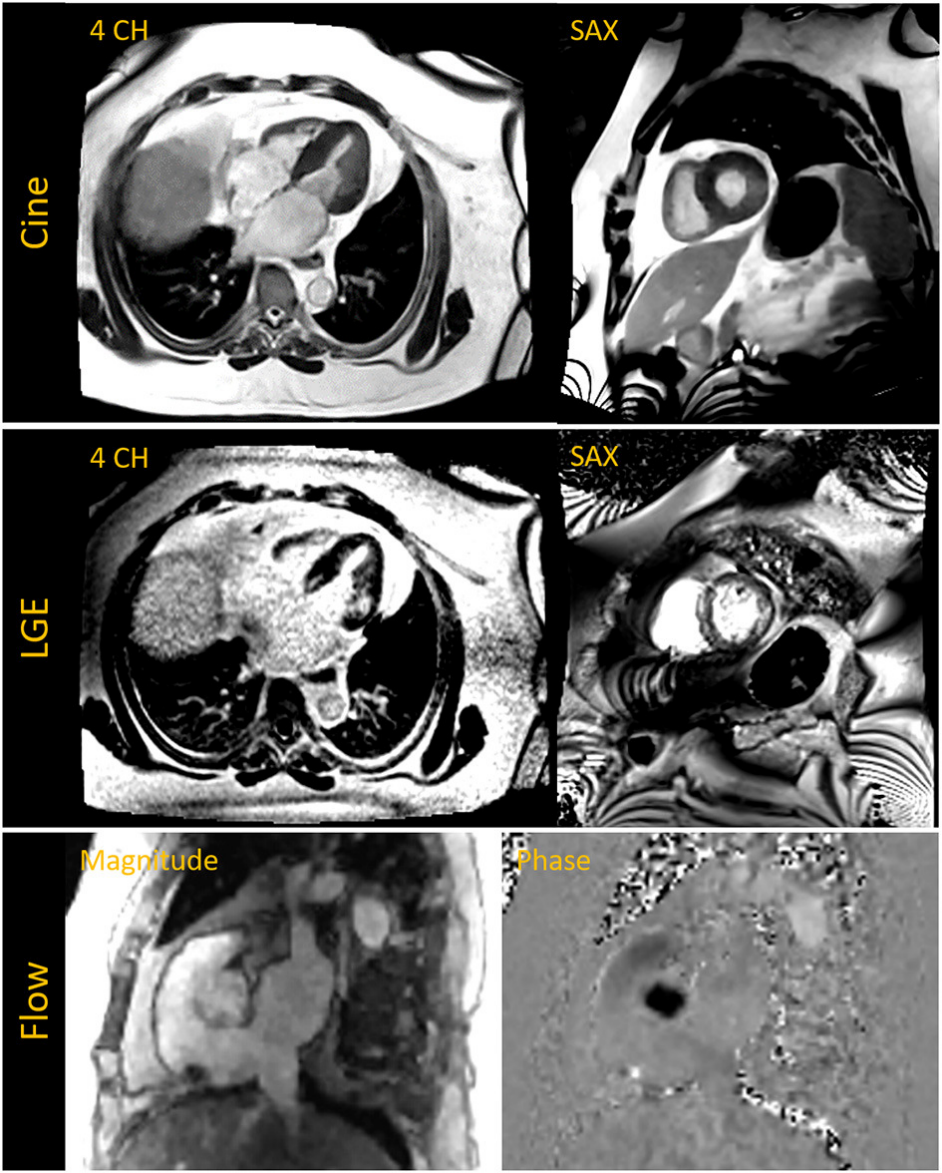
The **top row** shows breath-held segmented compressed sensing (CS) based cine images acquired in the four chamber (4 CH) and short axis (SAX) view in a patient with a BMI of 57 kg/m^2^ and who was previously unable to complete a cardiac MR exam on a 70 cm bore system. Late gadolinium enhanced (LGE) images in the middle row depict fibrosis on the septum and lateral wall. The 4 CH was acquired with a free-breathing motion-corrected (MOCO) LGE sequence while the SAX image was acquired with a breath-held segmented method. The **bottom row** shows magnitude and phase images of the aortic root acquired with a CS based 2D phase contrast cine for flow assessment.

Having conducted these initial experiments to build the individual techniques required for a complete CMR exam, we performed pilot exams on two patients who were clinically indicated for a CMR evaluation. These patients (A and B), having a BMI of 48 and 57 kg/m^2^, respectively, had previously attempted to undergo a CMR exam on our standard 70 cm bore 1.5 T and 3 T systems. Physical discomfort and anxiety prevented Patient A from entering the scanner bore. Patient B was able to enter the bore but could not remain for the complete study. Breath-held segmented cine, CS 2D PC MR flow and LGE images were acquired at 0.55 T for the two exams. Both patients demonstrated normal biventricular systolic function with non-ischemic fibrosis evident from the LGE images. Figure 10 shows images from Patient B. A non-gated CE-MRA of the thoracic aorta was additionally acquired in Patient A showing a dilated thoracic aorta (Figure 9D).

Each of the two exams described here did not include all of the CMR components described in previous sections. However, they incorporated a variety of pulse sequences and serve as anecdotal examples of the potential for this novel 0.55 T, ultra-wide bore system to extend CMR service to patients currently constrained by standard MR system dimensions. Both patients reported feeling less anxious and more comfortable in the 80 cm bore, therefore increasing their compliance and ability to complete the exam.

## 10. Conclusion

We built and implemented the imaging techniques required for a comprehensive CMR imaging protocol on a novel, commercial 0.55 T whole body MRI system with limited gradient performance. This initial experience demonstrates that a comprehensive CMR imaging protocol is feasible on this system, thus paving the way for more extensive evaluation and comparison with higher field results in patients.

Prototype GRAPPA and CS-based techniques were implemented, and increased averages and slice thicknesses were utilized when possible to boost SNR, while meeting spatial and temporal resolution requirements for clinical imaging within a reasonable scan time. All techniques, especially those that employ image registration and motion correction, require additional investigation and optimization. Visual assessment from pilot exams in healthy volunteers, porcine models, and patients diagnosed with cardiac pathologies demonstrated an image quality in cine, flow, segmented LGE, and both non-contrast and contrast MRA protocols, that provides the confidence to move forward with systematic validation studies to establish diagnostic accuracy and sensitivity with respect to clinically standard high field systems.

We anticipate this future evaluation will lead to a robust and comprehensive clinical CMR protocol that can ultimately be utilized with clinical confidence in patient cohorts currently unable to undergo an exam on narrower bore, higher field systems due to severe obesity or claustrophobia.

## Data availability statement

The raw data supporting the conclusions of this article will be made available by the authors, without undue reservation.

## Ethics statement

The studies involving human participants were reviewed and approved by the Institutional Review Board of The Ohio State University. The patients/participants provided their written informed consent to participate in this study. The animal study was reviewed and approved by Institutional Animal Care and Use Committee of The Ohio State University.

## Author contributions

JV, NJ, and OPS performed the MRI studies including protocol optimization. NJ, DG, CC, YL, and YP contributed toward pulse sequence development and implementation on the scanner. MS and MK designed the animal experiments. JV, NJ, CC, NN, YH, and OPS drafted sections of the manuscript. JV drafted the figures. JV, NJ, MT, RA, YH, and OPS were involved in overall study design and image quality evaluation. All authors contributed to the article and approved the submitted version.
